# Rapid evaluation by lung-cardiac-inferior vena cava (LCI) integrated ultrasound for differentiating heart failure from pulmonary disease as the cause of acute dyspnea in the emergency setting

**DOI:** 10.1186/1476-7120-10-49

**Published:** 2012-12-04

**Authors:** Katsuya Kajimoto, Keiko Madeen, Tomoko Nakayama, Hiroki Tsudo, Tadahide Kuroda, Takashi Abe

**Affiliations:** 1 Division of Cardiology, Sensoji Hospital, Tokyo, Japan; 2 Division of Respiratory Medicine, Sensoji Hospital, Tokyo, Japan; 3 Division of Internal Medicine, Sensoji Hospital, Tokyo, Japan

**Keywords:** Acute dyspnea, Ultrasound device, Emergency department, Acute heart failure syndromes

## Abstract

**Background:**

Rapid and accurate diagnosis and management can be lifesaving for patients with acute dyspnea. However, making a differential diagnosis and selecting early treatment for patients with acute dyspnea in the emergency setting is a clinical challenge that requires complex decision-making in order to achieve hemodynamic balance, improve functional capacity, and decrease mortality. In the present study, we examined the screening potential of rapid evaluation by lung-cardiac-inferior vena cava (LCI) integrated ultrasound for differentiating acute heart failure syndromes (AHFS) from primary pulmonary disease in patients with acute dyspnea in the emergency setting.

**Methods:**

Between March 2011 and March 2012, 90 consecutive patients (45 women, 78.1 ± 9.9 years) admitted to the emergency room of our hospital for acute dyspnea were enrolled. Within 30 minutes of admission, all patients underwent conventional physical examination, rapid ultrasound (lung-cardiac-inferior vena cava [LCI] integrated ultrasound) examination with a hand-held device, routine laboratory tests, measurement of brain natriuretic peptide, and chest X-ray in the emergency room.

**Results:**

The final diagnosis was acute dyspnea due to AHFS in 53 patients, acute dyspnea due to pulmonary disease despite a history of heart failure in 18 patients, and acute dyspnea due to pulmonary disease in 19 patients. Lung ultrasound alone showed a sensitivity, specificity, negative predictive value, and positive predictive value of 96.2, 54.0, 90.9, and 75.0%, respectively, for differentiating AHFS from pulmonary disease. On the other hand, LCI integrated ultrasound had a sensitivity, specificity, negative predictive value, and positive predictive value of 94.3, 91.9, 91.9, and 94.3%, respectively.

**Conclusions:**

Our study demonstrated that rapid evaluation by LCI integrated ultrasound is extremely accurate for differentiating acute dyspnea due to AHFS from that caused by primary pulmonary disease in the emergency setting.

## Introduction

Acute dyspnea is one of the main reasons for admission to the emergency department (ED) [1]. Physicians working in the ED often need to make a rapid diagnosis and devise a treatment plan on the basis of limited clinical information [2,3]. In paticular, acute heart failue syndromes (AHFS) are challenging, since the clinical, radiographic, and laboratory parameters have variable diagnostic value because AHFS are a heterogeneous set of clinical syndromes [4]. Traditional diagnostic criteria for heart failure are based on the history, physical examination, and chest radiograph findings [5-8]. However, these criteria are often not very useful for ED patients because of only having intermediate accuracy, i.e., high specificity with lower sensitivity [9]. Bedside maneuvers and tests that deliver rapid and reliable results represent a cornerstone of ED diagnostics [1,2]. Recently, it was reported that detection of pulmonary interstitial edema by lung ultrasound evaluation of B-lines has a high diagnostic accuracy for differentiating cardiac-related acute dyspnea from that due to chronic obstructive pulmonary disease (COPD) or bronchial asthma in the ED [3,10-14]. However, it can be very challenging to differentiate AHFS from severe bilateral pneumonia, pulmonary fibrosis, acute lung injury, or acute respiratory distress syndrome (ARDS) by lung ultrasound alone, because B-lines are not specific for cardiogenic pulmonary edema despite being a very sensitive indicator [12,13,15]. In order to rapidly and accurately identify the etiology in patients with acute dyspnea, assessment of LV systolic function, the severity of valvular regurgitation, and the severity of volume overload is mandatory, not only to confirm the diagnosis of AHFS but also to help determine the optimal initial treatment [2,16-21]. To assess the severity of volume overload, it has been reported that the right atrial pressure can be estimated by measuring the diameter of the inferior vena cava (IVC) using echocardiography [22-24]. Recently, Gargani suggested that adding lung ultrasound to echocardiography (integrated cardiopulmonary ultrasound) could help to differentiate the main causes of acute dyspnea [13]. However, the usefulness of integrated ultrasound evaluation of the lungs, heart, and IVC for determining the etiology of acute dyspnea in the ED has not been adequately studied [25]. Therefore, we examined the screening potential of rapid evaluation by lung-cardiac-inferior vena cava (LCI) integrated ultrasound for differentiating AHFS from primary pulmonary disease in ED patients with acute dyspnea.

## Methods

### Patient population

The study protocol was approved by our local ethics committee. From March 2011 to March 2012, 90 consecutive patients admitted to the ED of our hospital with acute dyspnea were enrolled. Patients with acute coronary syndrome or chest injury were excluded from this study. In addition, patients who had acute dyspnea due to neither cardiac nor pulmonary cause were excluded from this study. Within 30 minutes of admission, all enrolled patients received conventional physical examination, rapid lung, cardiac, and inferior vena cava [IVC] ultrasound with a hand-held device (Vscan^®^), electrocardiography, blood tests (including brain natriuretic peptide assay), and chest X-ray in the emergency room. This study is being conducted in accordance with the principles of the Declaration of Helsinki. Written informed consent was obtained from the patient for publication of this report and any accompanying images.

### Rapid lung, cardiac, and IVC integrated ultrasound

**Figure 1:**
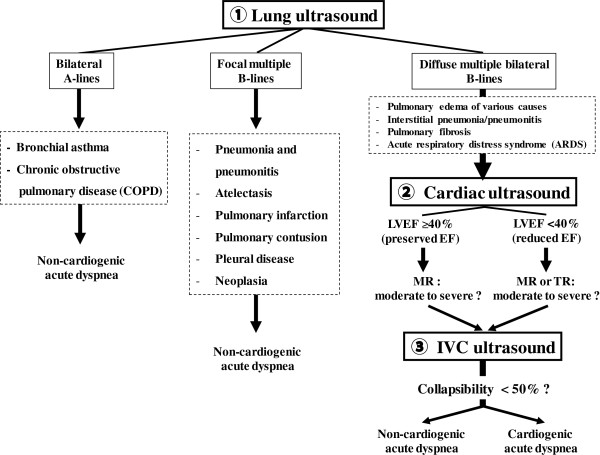
**Algorithm for the diagnosis of acute dyspnea based on the lung-cardiac-inferior vena cava integrated ultrasound.** LVEF = left ventricular ejection fraction; MR = mitral regurgitation; TR = tricuspid regurgitation; IVC = inferior vena cava.

**Figure 2:**
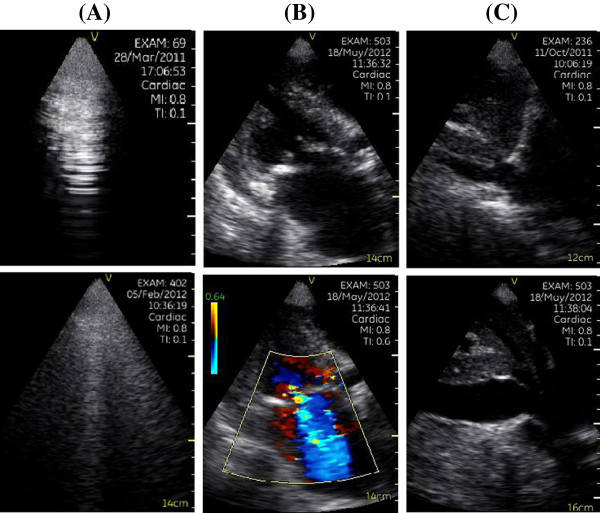
**Imaging of lung-cardiac-inferior vena cava(LCI)integrated ultrasound.** (**A**) Imaging of the lung ultrasound: Alines (upper) and B-lines (lower). (**B**) Imaging of the cardiac ultrasound: Apical long-axis view (upper) and moderate mitral regurgitation (lower). (**C**) Imaging of the inferior vena cava ultrasound: Collapsibility ≥ 50% (upper) and < 50% (lower).

The Vscan^®^ (GE Healthcare, Japan), a hand-held ultrasound device with a wide-bandwidth phased-array probe (1.7-3.5 MHz), was used in this study [26,27]. The investigators were unaware of the chest X-ray findings and clinical data of each patient. First, lung ultrasound was performed. Bilateral scanning of the anterior and lateral chest walls was done with the patient in the supine or sitting position. The correct scan was intercostal with maximum extension of the visual pleural line. The chest wall was divided into 8 areas (2 anterior and 2 lateral areas per side), and 1 scan was obtained for each area [3,14,28-30]. The anterior zone of the chest wall was designated from the sternum to the anterior axillary line and then was divided into upper and lower halves (from the clavicle to the third intercostal spaces and from the third space to diaphragm). The lateral zone was positioned from the anterior axillary line to the posterior axillary line and it was also divided into upper and lower halves. The investigators attempted to detect comet tail artifacts fanning out from the lung-wall interface and spreading to the edge of the screen, which was previously named B-lines [14,28]. According to the definition used in previous reports, lung ultrasound examination is positive if B-lines are found in two or more zones bilaterally of the eight zones assessed [3,28-30]. Lung ultrasound examination was always completed within 1 minute. Subsequently, cardiac ultrasound was performed. Global LV systolic function and the severity of mitral or tricuspid regurgitation were estimated visually from images acquired in standard cardiac views, particularly the apical long-axis view combined with the four-chamber view [16,17,25,31]. Preservation of the LV ejection fraction (EF) was defined as an estimated LVEF ≥40%, whereas a LVEF <40% indicated a reduced EF. Color scanning was also performed to assess flow across the mitral and tricuspid valves. Valvular regurgitation was semi-quantitatively assessed on a five-grade scale (none, trivial, mild, moderate, or severe), based on the width of the regurgitant jet at its origin estimated by visual inspection. A positive cardiac ultrasound examination meant that either a presence of moderate to severe mitral regurgitation (MR) in preserved EF subjects or a presence of moderate to severe MR or tricuspid regurgitation (TR) in reduced EF subjects was detected [19-21,32-34]. Finally, ultrasound evaluation of the IVC was examined within 2.0 cm of the IVC-RA junction. The maximum diameter (IVC max) was measured at the end-expiration and minimum diameter (IVC min) was measured at the end-inspiration [35]. The IVC collapsibility index (IVC-CI) was calculated as (IVC max-IVC min)/IVC max [35,36]. A positive IVC ultrasound examination, according to the definition in previous reports, required an IVC-CI <50% at baseline [23,24,35,37]. The duration of LCI-integrated ultrasound examination was always less than 3 minutes (Figure 1) [14]. The images of lung-cardiac-IVC integrated ultrasound were shown in Figure 2.

### Assay of brain natriuretic peptide (BNP)

Peripheral venous blood samples were obtained from each patient at admission, and then 5 ml of whole blood was placed into a prechilled vacuum tube containing EDTA for subsequent measurement of BNP. Immediately after blood sampling, each tube was placed on ice and centrifuged at 2,500 rpm and 4°C to obtain plasma. Then the plasma BNP level was measured by immunoradiometric assay using an antibody for human BNP (Shionogi Co. Ltd., Tokyo, Japan).

### Confirmation of diagnosis

The initial diagnosis was determined for each patient by one or two cardiologists, who performed lung-cardiac-IVC (LCI) integrated ultrasound evaluation within 3 minutes on each patient in the ED. Confirmation that acute dyspnea was due to a cardiac etiology (AHFS) was based on a positive lung ultrasound examination combined with abnormal findings on either cardiac or IVC ultrasound in the ED (Figure 1). To determine the final diagnosis, two cardiologists and one pneumologist, who were blinded to the results of the LCI integrated ultrasound at admission, independently reviewed each patient’s medical records and classified them as having acute dyspnea due to AHFS, a history of HF but acute dyspnea due to a non-cardiac cause, or non-cardiac acute dyspnea. Confirmation of AHFS was based on the generally accepted Framingham criteria (two major criteria or one major and two minor criteria), with corroborative information including the medical history, hospital course (response to diuretics and vasoactive agents, or results of hemodynamic monitoring), and routine laboratory test data (including BNP) [5,38]. The category of pulmonary acute dyspnea included pulmonary embolism and primary lung diseases (pneumonia, asthma, COPD, pulmonary fibrosis, or acute respiratory distress syndrome), with or without underlying LV systolic dysfunction but with no evidence of decompensated HF at admission.

### Statistical analysis

Analyses were performed with SAS 9.1 software (SAS Institute, Cary, North Carolina). Quantitative variables were compared by using Student’s *t*-test, and dichotomous variables were compared with the chi-square test. The area under the receiver operating characteristic curves and the Youden index were calculated to define the optimum cut-off value of BNP for differentiating AHFS from pulmonary disease. The sensitivity, specificity, negative predictive value, and positive predictive value were calculated according to standard definitions. Two-tailed P values of less than 0.05 were considered to indicate a statistically significant difference. All analyses were performed by an independent biostatistics center (Statz Institute, Inc., Tokyo, Japan).

## Results

Among 90 consecutive patients with acute dyspnea (45 females, 78.1 ± 9.9 years), the final diagnosis was acute dyspnea due to AHFS in 53 patients, acute dyspnea due to a pulmonary cause despite a history of heart failure in 18 patients, and acute dyspnea due to a pulmonary cause in 19 patients. Cardiogenic acute dyspnea (AHFS) was due to ischemic heart disease, hypertensive heart disease, valvular heart disease, and idiopathic dilated cardiomyopathy in 10, 29, 6, and 8 patients, respectively. Non-cardiogenic acute dyspnea was due to asthma or COPD, pneumonia, pulmonary fibrosis, and ARDS in 16, 9, 7, and 5 patients, respectively. The main clinical characteristics of the patients stratified according to their final diagnoses are shown in Table 1. When patients with AHFS-related acute dyspnea were compared to those with pulmonary-related acute dyspnea, both groups were of a similar age and had a similar rate of prior hospitalization for HF, but a history of hypertension was more common in the AHFS group. The plasma BNP level was significantly higher in the AHFS group than in the pulmonary group. On the other hand, C-reactive protein was significantly higher in the pulmonary group than in the AHFS group.

**Table 1:** Baseline characteristics in overall patients and according to final diagnosis

	**All patients (n = 90)**	**AHFS group (n = 53)**	**Pulmonary group (n = 37)**	**P value**
Mean age, yrs	78.1 ± 9.9	77.7 ± 10.3	78.6 ± 9.2	0.662
Female sex	45 (50.0)	29 (54.7)	16 (43.2)	0.284
Medical history				
Prior hospitalization for heart failure	42 (46.7)	27 (50.9)	15 (40.5)	0.330
Hypertension	51 (56.7)	38 (71.7)	13 (35.1)	<0.001
Dyslipidemia	20 (22.2)	16 (30.2)	4 (10.8)	0.029
Diabetes	11 (12.2)	7 (13.2)	4 (10.8)	0.733
Chronic obstructive pulmonary disease	24 (26.7)	13 (24.5)	11 (29.7)	0.583
History of atrial fibrillation	22 (24.4)	12 (22.6)	10 (27.0)	0.634
Medications prior to admission				
Loop diuretic	47 (52.2)	31 (58.5)	16 (43.2)	0.154
Spironolactone or Eplerenone	31 (34.4)	22 (41.5)	9 (24.3)	0.091
ACE inhibitor or ARB	45 (50.0)	33 (62.3)	12 (32.4)	0.005
Beta-blocker	27 (30.0)	17 (32.1)	10 (27.0)	0.607
Calcium channel blocker	13 (14.4)	9 (16.9)	4 (10.8)	0.413
Digoxin	8 (8.9)	6 (11.3)	2 (5.4)	0.332
Laboratory data				
Brain natriuretic peptide, pg/ml	461.1 ± 451.9	622.0 ± 505.3	230.7 ± 208.2	<0.001
Blood urea nitrogen, mg/dl	25.6 ± 14.3	26.0 ± 15.2	24.8 ± 13.0	0.701
Serum creatinine, mg/dl	1.07 ± 0.51	1.12 ± 0.58	0.99 ± 0.36	0.203
C-reactive protein, mg/dl	3.64 ± 5.73	1.96 ± 3.17	6.05 ± 7.52	<0.001
Symptoms on admission				
Orthopnea	42 (46.7)	25 (47.2)	17 (45.9)	0.901
Paroxysmal nocturnal dyspnea	40 (44.4)	28 (52.8)	12 (32.4)	0.055
Peripheral edema	48 (53.3)	37 (69.8)	11 (29.7)	<0.001
Signs on admission				
Rales	56 (62.2)	37 (69.8)	19 (51.3)	0.075
Wheezing	38 (42.2)	20 (37.7)	18 (48.6)	0.302
Jugular venous distension	25 (27.8)	19 (35.8)	6 (16.2)	0.041
S3	19 (21.1)	15 (28.3)	4 (10.5)	0.045
Ultrasound findings				
Pleural effusion	18 (20.0)	14 (26.4)	4 (10.5)	0.069
Lung consolidation	10 (11.1)	1 (1.9)	9 (24.3)	<0.001
Reduced EF (LVEF <40%)	22 (24.4)	16 (17.8)	6 (16.2)	0.129
MR ≥ moderate	41 (45.6)	41 (77.3)	0 ( 0.0)	<0.001
TR ≥ moderate	38 (42.2)	34 (64.5)	4 (10.5)	<0.001
IVC collapsibility <50%	51 (56.7)	44 (83.0)	7 (18.9)	<0.001

Values are shown as the no. (%), mean ± SD. AHFS = acute heart failure syndromes; ACE = angiotensin-converting enzyme; ARB = angiotensin receptor blocker. LV = left ventricular; EF = ejection fraction; MR = mitral reguragitation; TR = tricuspid regurgitation; IVC = inferior vena cava.

### Relation between plasma BNP and final diagnosis

Patients with acute dyspnea due to AHFS had a BNP level of 622.0 ± 505.3 pg/ml, which was significantly higher than the BNP level of 230.7 ± 208.2 pg/ml in patients with a final diagnosis of pulmonary disease (p < 0.001). In the group with acute dyspnea due to pulmonary disease, the 18 patients with a history of heart failure had a significantly higher BNP level compared to the 19 patients without a history of heart failure (396.7 ± 176.5 vs. 73.4 ± 59.6 pg/ml, p < 0.001). The BNP level of patients with a history of heart failure and dyspnea due to pulmonary disease showed no significant difference from that of the patients with acute dyspnea due to AHFS (396.7 ± 176.5 vs. 622.0 ± 505.3 pg/ml; p = 0.069). In addition, the BNP level of patients with ARDS (n = 5) showed no significant difference from that of patients with acute dyspnea due to AHFS (369.5 ± 246.3 vs. 622.0 ± 505.3 pg/ml; p = 0.277). The ability of BNP to differentiate AHFS from pulmonary disease was assessed by ROC analysis. The area under the ROC curve for differentiating AHFS from pulmonary disease with BNP was 0.750 (95% confidence interval: 0.698 to 0.804). A BNP value of 663.2 pg/ml had a sensitivity of 37.0%, specificity of 97.2%, negative predictive value of 50.7%, and positive predictive value of 95.2% for differentiating AHFS from pulmonary disease.

### Lung-cardiac-inferior vena cava (LCI) integrated ultrasound

In Table 2, the sensitivity, specificity, negative predictive value (PV), positive PV, and total accuracy for differentiating AHFS from pulmonary disease in emergency patients with acute dyspnea are presented for Framigham criteria (two major or one major and two minor criteria), BNP (cut-off value: 100 pg/mL), lung ultrasound, both lung ultrasound and BNP (cut-off value: 100 pg/mL), IVC collapsibility (cut-off value: 50%), either MR or TR (≥ moderate), both preserved EF and MR (≥ moderate), both reduced EF and either MR or TR (≥ moderate), and LCI integrated ultrasound. Comparing these methods showed that LCI integrated ultrasound had the highest specificity (91.9%), negative PV (91.9%), positive PV (94.3%), and total accuracy (93.3%). While lung ultrasound alone had the highest sensitivity (96.2%), its specificity was much lower (54.0%). A reduced EF showed the lowest sensitivity and lowest total accuracy (26.4% and 51.1%, respectively), while BNP at a cut-off value of 100 pg/mL had the lowest specificity (35.1%).

**Table 2:** Plasma BNP, lung ultrasound alone or combined with BNP, cardiac findings, and the LCI integrated ultrasound for diagnosis of AHFS

	**Sensitivity (%)**	**Specificity (%)**	**NPV (%)**	**PPV (%)**	**Accuracy (%)**
BNP ≥100 pg/ml	92.4	35.1	76.4	67.1	68.8
Framingham criteria*	79.2	56.7	65.6	64.6	70.0
Lung ultrasound alone	96.2	54.0	90.9	75.0	78.8
Both Lung ultrasound and BNP (≥100 pg/ml)	88.6	67.6	80.6	79.8	80.0
Reduced EF (LVEF <40%)	26.4	86.5	45.1	73.7	51.1
MR or TR ≥ moderate	92.4	81.0	88.2	87.5	87.7
IVC collapsibility <50%	83.0	81.1	76.9	86.3	82.2
Both preserved EF and MR ≥ moderate	56.7	100.0	61.6	100.0	67.0
Both reduced EF and either MR or TR ≥ moderate	30.1	94.5	48.6	88.9	56.7
Lung-cardiac-inferior vena cava (LCI) integrated ultrasound	94.3	91.9	91.9	94.3	93.3

* Two major or one major and two minor criteria. BNP = brain natriuretic peptide; LCI = lung-cardiac-inferior vena cava; AHFS = acute heart failure syndromes; NPV = negative predictive value; PPV = positive predictive value; LVEF = left ventricular ejection fraction; IVC = inferior vena cava; MR = mitral regurgitation; TR = tricuspid regurgitation.

## Discussion

The present study demonstrated that rapid evaluation by lung-cardiac-inferior vena cava (LCI) integrated ultrasound has a higher diagnostic accuracy for differentiating acute dyspnea due to AHFS from pulmonary acute dyspnea (including COPD/asthma, pulmonary fibrosis, and ARDS) compared with lung ultrasound either alone or in combination with plasma BNP assay. These findings suggest that LCI integrated ultrasound has become a fundamental tool for diagnostic evaluation of patients with acute dyspnea and selection of early treatment in the emergency setting.

Rapid and accurate diagnosis and management can be lifesaving for patients with acute dyspnea [39]. However, making a differential diagnosis and selecting early treatment for patients with acute dyspnea in the ED is a clinical challenge that requires complex decision-making in order to achieve hemodynamic balance, improve functional capacity, and decrease mortality and the length of hospital stay [40]. Methods for evaluation of emergency patients with possible AHFS include the history, physical examination, chest radiography, 12-lead electrocardiography, and measurement of BNP or N-terminal pro-BNP [5-10]. Among these methods, chest radiography is a cornerstone in the diagnostic evaluation of acute dyspnea. Although chest radiography serves a vital role in the evaluation of patients with acute dyspnea, including the identification of various causes, the lack of radiographic signs of congestion does not exclude AHFS [2,41]. Recently, BNP and N-terminal pro-BNP have been studied extensively and are frequently used in clinical practice. However, some recent randomized trials on the use of BNP to aid in diagnosis or serial BNP levels to dictate therapy in the acute setting found no improvement of diagnostic accuracy or important clinical outcomes because age, sex, and renal dysfunction have an impact on natriuretic peptide levels and need to be considered when test results are interpreted [42,43]. Also, patients with a history of decompensated HF can have chronically elevated BNP or N-terminal pro-BNP levels, making the test inconclusive. In addition, it was reported that BNP does not reliably distinguish ARDS from AHFS [44]. In our study, the BNP level of patients with a history of heart failure who had dyspnea due to pulmonary disease or ARDS showed no significant difference compared to that of patients with acute dyspnea due to AHFS, a finding that is in agreement with prior reports [42,43]. Therefore, among patients with acute dyspnea (including those with a history of heart failure and those with ARDS), the baseline BNP level alone could have various limitations for making a differential diagnosis in the emergency setting, and further research is needed to address this issue.

B-lines assessed by lung ultrasound have been proposed as an easy alternative diagnostic tool for monitoring pulmonary congestion in AHFS patients [28]. Recently, it was reported that B-lines alone or B-lines combined with N-terminal pro-BNP show a high diagnostic accuracy for differentiating AHFS-related acute dyspnea from that due to COPD/asthma in the ED [3,41]. However, it is impossible to differentiate AHFS from bilateral pneumonia, pulmonary fibrosis, or ARDS by lung ultrasound alone, because although B-lines are a very sensitive sign of cardiogenic pulmonary edema, this sign is not specific [13]. However, in the present study, the lung ultrasound in two patients with pure right-sided heart failure, which was not in association with left-sided heart failure, showed a false negative, suggesting that B-lines may not be sensitive for pure right-sided heart failure. Recently, Gargani has suggested that addition of lung ultrasound to echocardiography provides additive information about pulmonary involvement [13]. Furthermore, Kimura et al. has reported the usefulness of cardiopulmonary-limited ultrasound examination consisting of only 4 ultrasound views, such as LV systolic dysfunction, left atrial enlargement, IVC, and B-lines, for the diagnostic accuracy and prognostic information, although they did not evaluate a diagnostic accuracy for differentiating acute dyspnea due to AHFS from that caused be primary pulmonary disease [45]. On the basis of these available reports and our findings, it is suggested that LCI integrated ultrasound assists with the rapid and accurate diagnosis and treatment of acute dyspnea in the emergency setting.

Our study had several limitations. First, this was a single-center investigation of a small patient population. Second, assessment of diastolic dysfunction and quantitative analysis of valvular heart disease could not be done with the hand-held ultrasound device employed in this study. Therefore, complete evaluation of acute dyspnea in patients requires comprehensive standard echocardiography after LCI ultrasound evaluation. Third, we could not evaluate the extravascular lung water in AHFS patients because we did not examine the number of B-lines. Therefore, further prospective investigation to evaluate the extravascular lung water by a hand-held device for patients with acute dyspnea in the emergency setting is needed. Fourth, training is needed to interpret the findings of LCI ultrasound.

In conclusion, our study demonstrated that rapid evaluation by lung-cardiac-IVC (LCI) integrated ultrasound has a higher accuracy for differentiating AHFS-related acute dyspnea from pulmonary-related acute dyspnea compared with lung ultrasound alone or lung ultrasound combined with BNP. These findings suggest that LCI integrated ultrasound is a useful tool to expedite the evaluation of patients with acute dyspnea before initiating treatment in the ED. However, further research will be needed to provide more insight into the impact of LCI integrated ultrasound using a portable ultrasound device on diagnosis and decision making in the ED.

## Abbreviations

AHFS: Acute heart failure syndromes; LCI: Lung-cardiac-inferior vena cava; IVC: Inferior vena cava; ED: Emergency department.

## Competing interests

The authors declare that they have no competing interests.

## Authors’ contributions

KK, TN, and TA conceived this study. KK and KM performed lung-cardiac-inferior vena cava ultrasound in patients with acute dyspnea. KK, HT, and TK participated in the study design and coordination an helped to draft the manuscript. All authors read and approved the final manuscript.
